# Birds, Bats or Climate? Eucalypt Floral Traits Reflect Pollination Over Abiotic Environment

**DOI:** 10.1002/ece3.71449

**Published:** 2025-06-11

**Authors:** R. E. Stephens, H. Sauquet, B. Laugier, C. R. Gosper, R. V. Gallagher

**Affiliations:** ^1^ School of Natural Sciences Macquarie University Ryde New South Wales Australia; ^2^ National Herbarium of NSW Botanic Gardens of Sydney Mt Annan New South Wales Australia; ^3^ Ecology & Evolution Research Centre University of New South Wales Sydney New South Wales Australia; ^4^ Hawkesbury Institute for the Environment Western Sydney University Richmond New South Wales Australia; ^5^ Biodiversity and Conservation Science Department of Biodiversity, Conservation and Attractions Perth Western Australia Australia

**Keywords:** floral traits, functional biogeography, macroecology, macroevolution, pollination

## Abstract

Flowers and their traits vary greatly across species, influenced by biotic and abiotic environmental variation. We explore the relative effects of pollination and abiotic environment on flower size and colour in a species‐rich tree clade (eucalypts: *Eucalyptus*, *Corymbia* and *Angophora*). Most eucalypt flowers are small and white‐cream with generalised pollination systems. Though larger, more colourful (i.e., red, pink, orange, yellow or green) eucalypt flowers occur more frequently in southwest Australia, it remains unclear what environmental factors contribute to this pattern. We extracted bud size (as a proxy for flower size) and flower colour (as white‐cream or colourful) for 798 eucalypt species from online floras. We assessed three measures of vertebrate pollination environment—flower‐visiting bird species richness, flower‐visiting marsupial presence/absence, and flower‐visiting bat presence/absence—and three measures of abiotic environment—mean annual temperature, mean annual precipitation, and soil available phosphorus. We show that flower size and colour have evolved in tandem in eucalypts and are not well predicted by contemporary climate or soil environments. Instead, pollination environment, and particularly the absence of flower‐visiting bats, was the strongest predictor of eucalypt flower size and colour. Larger, more colourful eucalypt flowers may have evolved to attract bird pollinators in landscapes where bats are not available to carry pollen long distances. Small, white‐cream eucalypt flowers, conversely, may represent a successful generalist pollination syndrome where insects, bats, birds, and/or marsupials all contribute to pollination. Continental‐scale patterns of floral trait variation thus reflect macroecological patterns in pollinator availability, revealing elements of the biotic environment that may shape plant reproductive strategies.

## Graphical abstract

Flower colour and size have evolved in tandem in the eucalypts, with larger and more colourful flowers arising multiple times in southwest Western Australia. Eucalypt flower size and colour are more strongly associated with the vertebrate pollination environment—especially the absence of flower‐visiting bats—than with climatic or soil conditions.

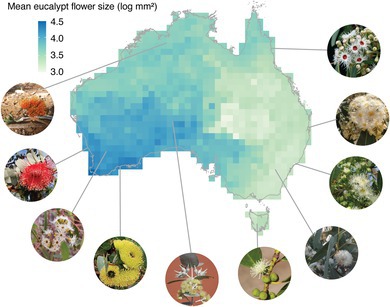

## 1 Introduction

Most flowers, for most of their evolutionary history, have been pollinated by animals (Friis et al. 2011; Stephens et al. 2023). Animal pollinators have thus shaped the wide array of floral features that make flowering plants so diverse (Ollerton et al. 2019; van der Niet and Johnson 2012). While scientists have focussed on the importance of animal pollinators to floral forms since the 1700s (reviewed in Waser 2006), research attention has recently turned to the influence that the abiotic environment also has on floral traits (Caruso et al. 2019; Dalrymple et al. 2020; E‐Vojtkó et al. 2020; Roddy et al. 2021). Contemporary studies have described large‐scale patterns of variation in floral traits, often tied to variation in abiotic factors such as climate (Song et al. 2022; Stephens et al. 2022; Wang et al. 2023).

Flower size and colour are two traits expected to respond to both biotic and abiotic pressures. Flower size can be strongly influenced by pollinator size via size matching (Fenster et al. 2004; Naghiloo et al. 2021), but can also be influenced by resource availability, with larger flowers in environments with higher temperature and precipitation (Lambrecht and Dawson 2007; Roddy et al. 2021). Similarly, flower colours often match the visible spectrum seen by pollinators (Burd et al. 2014; Fenster et al. 2004; Garcia et al. 2022) yet can also be shaped by environmental stress, with more saturated colours found in hot or dry conditions (Dalrymple et al. 2020; Strauss and Whittall 2006). The links between soil nutrients and flower size and colour are less well explored but are often mixed and mediated by pollinators (Carvalheiro et al. 2021).

Abiotic and biotic influences on flower size and colour are easier to isolate in specialised pollination systems but can overlap in generalised pollination, where multiple pollinator types visit the same flowers. In generalised systems, different pollinators may exert contrasting or diffuse selection on floral traits, making evolutionary relationships harder to detect (Dellinger 2020; Ollerton 2017). This can lead to mixed or context‐dependent trait evolution across environmental gradients, especially in widespread taxa exposed to diverse communities of pollinators and abiotic conditions. Given generalised pollination is arguably more common than specialised pollination (Dellinger 2020; Waser et al. 1996), though both do exist on a continuum (Ollerton 2017), understanding the relative contributions of the biotic and abiotic environment in more generalised systems is essential to predicting large‐scale floral trait–environment relationships.

Here we explore the relationship between the biotic and abiotic environment and flower size and colour in a widespread, generalist‐pollinated tree clade, the eucalypts. Eucalypts (genera *Eucalyptus*, *Corymbia* and *Angophora*, family Myrtaceae) are highly diverse with over 800 species in total and are the dominant tree in most Australian forest ecosystems (Thornhill et al. 2019, Figure S1). Eucalypts are an ideal system for studying variation in flower size due to their highly consistent floral architecture (CANBR et al. 2020; House 1997). Eucalypt flowers are actinomorphic and pentamerous with numerous stamens as the primary visual signal for pollinators (House 1997). As preferential outcrossers, eucalypts produce large quantities of nectar to attract a range of potential pollinators (Southerton et al. 2004). Most eucalypts flower over spring and summer (September–February), though flowering season and intensity vary with abiotic conditions, and flowers typically remain open and accessible for several days to weeks, allowing visitation by both diurnal and nocturnal pollinators (CANBR et al. 2020; House 1997).

While most eucalypt flowers are relatively small (1.5–2.5 cm wide in ca. 75% of species), some eucalypt flowers can be very large, up to 10 cm across in some instances (e.g., *Eucalyptus macrocarpa* ). Large eucalypt flowers are often more colourful (i.e., non‐white‐cream in human colour vision), and tend to occur in southwestern Australia (House 1997). Whether this pattern is general across the eucalypts and what drives its occurrence are yet to be established, however, and several exceptions do exist, with large, white‐cream flowers in some species (e.g., *Eucalyptus aquilina*), and species with large colourful flowers also found in central and northern Australia (e.g., *Eucalyptus pachyphylla* , *Corymbia ptychocarpa*). It is also yet to be tested whether the relationship between flower size, colour and westerly distribution is an evolutionary coincidence confined to one or a few related clades, or a case of correlated evolution, perhaps in response to a difference in biotic or abiotic environment in the west of Australia.

**FIGURE 1 |:**
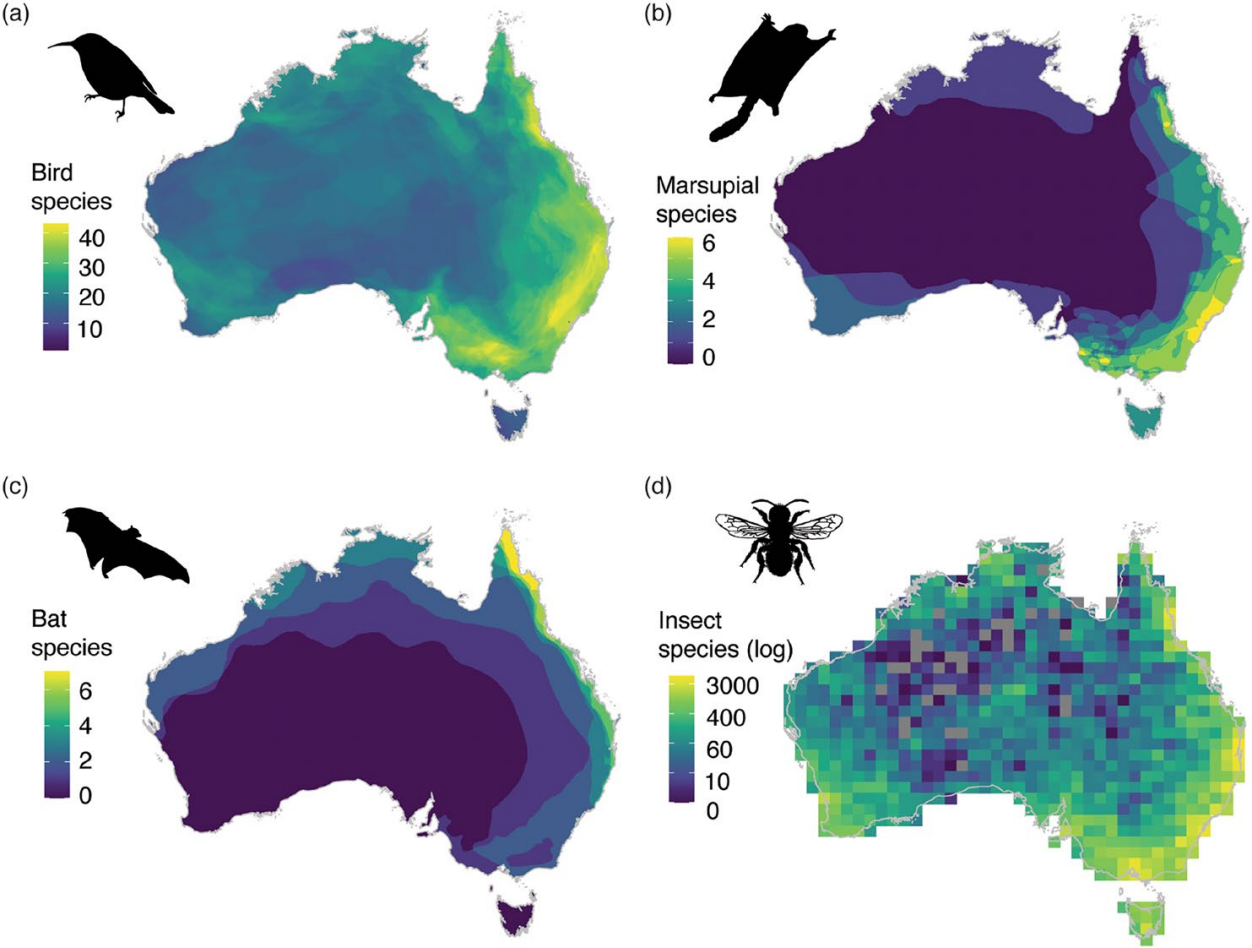
Species richness of eucalypt pollinator groups including flower‐visiting birds (a), marsupials (b), bats (c) and insects (log scale) (d) across Australia. Species silhouettes from phylopic.org show Eastern Spinebill (a), Sugar Glider (b), Grey‐headed Flying Fox (c) and a leaf cutter bee (d). Bird, marsupial and bat species richness derived from species range maps (see Methods). Flower‐visiting insect species richness was calculated using occurrence data from the Atlas of Living Australia using the R package galah (Westgate et al. 2023, see Note S1 for details). Patterns of species richness in the insect map likely reflect sampling biases more than patterns of actual species richness, given the lack of insect occurrence data in many locations across Australia.

Eucalypts are visited and pollinated by a wide range of insects, birds, bats, and marsupials (Armstrong 1979; Bacles et al. 2009; Hingston et al. 2004b; House 1997; Southerton et al. 2004). While larger, more colourful flowers have been linked to more specialised bird pollination in several species (Bezemer et al. 2019; Hopper and Moran 1981; House 1997), it remains unclear what environmental factors may have driven this specialisation. Birds are abundant and active flower visitors across Australia, so the availability of bird pollinators is unlikely to be a limiting factor (Ford et al. 1979, Figure 1a). Areas of high bird diversity may support greater diversity in eucalypt flower size and colour, however, as floral traits adapt to a wider range of visitor morphologies, physiologies, and behaviours (Fenster et al. 2004). Much less is known about the distribution of insect pollinators across Australia (Braby 2018; Saunders et al. 2021), but flower‐visiting insects should be available in most Australian landscapes when plants are flowering (Armstrong 1979; House 1997, Figure 1d). Marsupial pollinators (gliders and small possums) are not available in all parts of Australia (Figure 1b) but do not have powered flight and are thus likely less effective outcross pollinators of eucalypt trees (Armstrong 1979; House 1997). Pteropodidae bats do contribute to outcross pollination in eucalypts, especially in eastern and northern Australia where flying‐foxes migrate with flowering events (Armstrong 1979; Bacles et al. 2009; Bradford et al. 2022). Flower‐visiting bats are largely absent from central and southwest Australia, creating regional variation in their potential influence on eucalypt flower evolution (Figure 1c; Milne et al. 2023).

**FIGURE 2:**
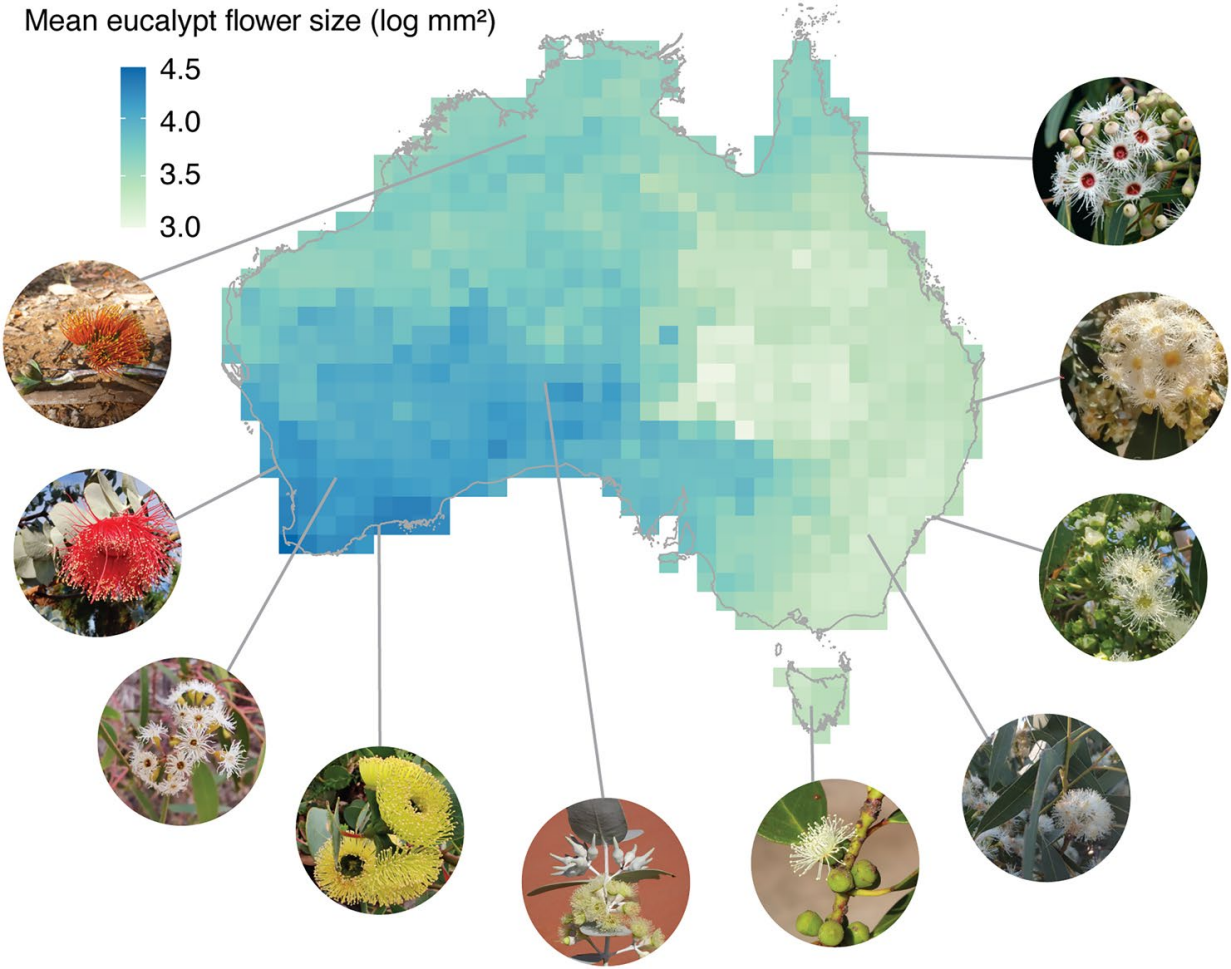
Mean eucalypt flower size (log mm^2^) in 100 × 100 km grid cells across Australia. Images exemplify the variety of sizes and colour in eucalypt flowers, with lines connecting species to the approximate midpoint of their geographic range. Eucalypt species clockwise from top right, with subgenus and section in parentheses: *Corymbia rhodops* (Corymbia, Bruce Gray via EUCLID), *Eucalyptus microcorys* (Alveolata), *Angophora costata* (Angophora), *Eucalyptus blakelyi* (Symphyomyrtus—Exsertaria), *Eucalyptus subcrenulata* (Symphyomyrtus—Maidenaria, Dean Nicolle), *Eucalyptus wyolensis* (Symphyomyrtus—Bisectae, Dean Nicolle), *Eucalyptus preissiana* (Eucalyptus), *Eucalyptus yilgarnensis* (Symphyomyrtus—Bisectae), *Eucalyptus rhodantha* (Symphyomyrtus—Bisectae) and *Eucalyptus miniata* (Eudesmia). All photos by Ruby Stephens unless otherwise credited.

**FIGURE 3:**
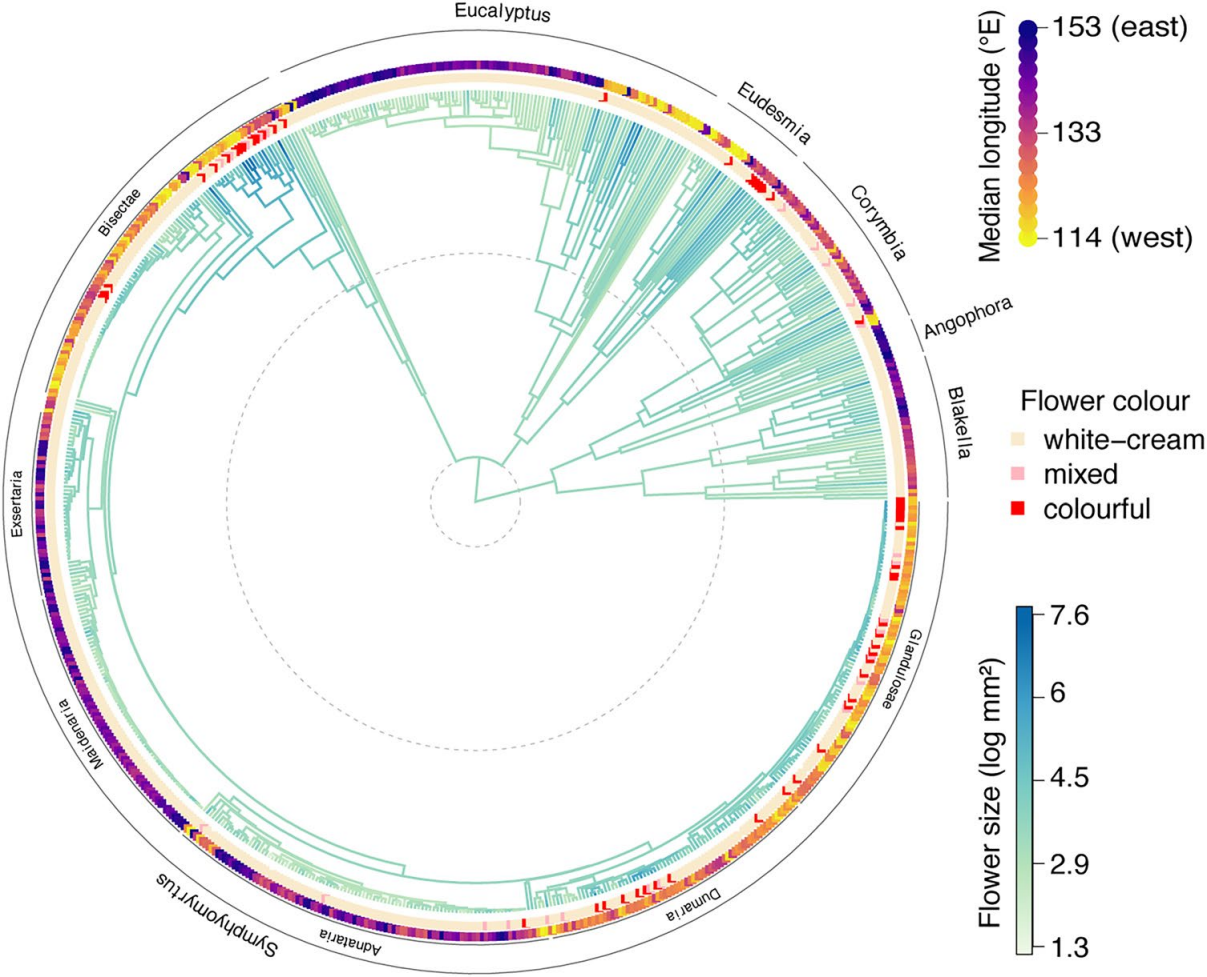
Species‐level time‐calibrated phylogeny of the eucalypts from Thornhill et al. (2019), with phylogeny branches coloured by simulated evolution of eucalypt flower size (log mm^2^). Tip points indicate species flower colour, and species median longitude from eastern Australia (blue) to western Australia (orange‐yellow). Labels indicate major subgenera and sections, though note these are not fully monophyletic in the current phylogeny. Dotted lines in the background indicate the Eucalyptae crown age ca. 52 million years ago (Ma) and the beginning of the Neogene period 23 Ma.

In this study, we use macroecological and phylogenetic analyses to study associations among eucalypt flower size, approximated by bud size; flower colour, approximated by a binary of white‐cream or colourful in human colour vision; and features of both the biotic (pollination) and abiotic (climate and soil variation) environment. We use a broad human colour vision binary to make use of available data for a large number of species, though we acknowledge that high‐resolution reflectance data and pollinator vision modelling could reveal different patterns (e.g., Dalrymple et al. 2020; Garcia et al. 2022). Using gridded data on animal species ranges, we develop three measures of vertebrate pollination environment: (1) the species richness of flower‐visiting birds, as a proxy for bird pollinator availability (Figure 1a); (2) the presence/absence of flower‐visiting marsupials (Figure 1b), and (3) the presence/absence of flower‐visiting bats (Figure 1c). We focus on the vertebrate pollination environment given significant knowledge gaps in the taxonomy and distribution of Australian insect pollinators (Braby 2018, Figure 1d). We develop independent models of how both flower size and flower colourfulness vary with these biotic predictors and compare the predictive power of these models to those fit with gridded data on mean annual temperature (MAT, °C), mean annual precipitation (MAP, mm), and available soil phosphorus (P_avail_, mg/kg). We selected phosphorus because it is a key limiting nutrient in many Australian soils with a strong influence on Australian plant evolution and has known effects on plant growth and investment (Fonseca et al. 2000; Handreck 1997). We assess the relative effect of each of these measures of vertebrate pollination (birds, marsupials, bats) and abiotic (MAT, MAP, P_avail_) environment on flower size and colourfulness across the eucalypts and use phylogenetic comparative methods to explore the relationship between eucalypt flower size, colourfulness, and distribution.

## 2 Materials and Methods

All data extraction, processing, and analysis was completed in R version 4.3.0 (R Core Team 2023) using packages including the tidyverse collection (Wickham et al. 2019), terra version 1.7‐55 (Hijmans et al. 2022), sf version 1.0‐14 (Pebesma 2018), ape version 5.7‐1 (Paradis and Schliep 2019) and phytools version 2.0‐3 (Revell 2024). All data and analysis code are available at https://doi.org/10.5281/zenodo.10578823.

### 2.1 Trait Data

Eucalypt trait data was extracted from an online flora of the eucalypts, EUCLID (CANBR et al. 2020) using methods described by Coleman et al. (2023). Briefly, regular expressions were used to extract trait values from downloaded species descriptions for traits including flower colour and bud dimensions. All extracted values were error‐checked, and for 12 missing species, supplemented from original species descriptions (Hill and Johnson 1995, 2001; Hill and Stanberg 2002; Johnson and Hill 1990, 1991; Nicolle 2001; Watson et al. 1987). Taxonomy was aligned to the Australian Plant Census using the R package APCalign (Wenk et al. 2024), and trait values were summarised at the species‐level for *n* = 798 species.

Flower colour was categorised as white‐cream (*n* = 696 species), colourful (*n* = 65 species), or mixed for polymorphic taxa with either a combination of white‐cream and other colours within the flower or variable flower colouration across their range (*n* = 28 species). Colourful flowers included any flowers where the dominant colour of the filaments and hypanthium was red, pink, orange, yellow, or green. Flower dimensions are rarely if ever reported in eucalypt taxonomic descriptions, whereas bud dimensions are consistently reported. Given all parts of the flower are contained within the mature bud, we used bud size as a proxy for flower size. We assessed the appropriateness of this proxy by confirming the strong relationship between bud and flower dimensions, as measured on a range of eastern (longitude 138.67°–153.63° E) and western (longitude 112.93°–123.8° E) Australian eucalypts in the field (Figure S2; *n* = 23 species, ordinary least squares (OLS) regression *p* < 0.001, *R* ^2^ = 0.88). Species mean bud size in mm^2^ was calculated from bud dimensions by taking the mean of minimum and maximum values for length and width, then multiplying length × width, giving bud size (hereafter, flower size) for *n* = 791 species. Flower size data were right‐skewed, with an interquartile range of 22–70 mm^2^ but many outliers above this and a maximum of 2588 mm^2^ (*Eucalyptus brandiana*). Given this distribution, flower size was log transformed for all analyses.

### 2.2 Environment and Distribution Data

To calculate measures of typical environmental conditions across the geographic range of each eucalypt species, we used herbarium occurrences downloaded from the Australasian Virtual Herbarium and manually cleaned by comparison against known species ranges in EUCLID (CANBR et al. 2020). To avoid possible spatial bias, we thinned occurrences to remove any within 5 km of another occurrence of the same species using the R package spThin (Aiello‐Lammens et al. 2015). Five kilometres was chosen as the cut‐off as it is the maximum resolution of environmental variables used.

Abiotic environmental variables assessed were MAT (°C), MAP (mm), and mean soil available phosphorus (P_avail_, mg/kg). Given that most eucalypts are hypothesised to have diversified in the Plio‐Pleistocene (Thornhill et al. 2019), we used contemporary environmental variables as a proxy for the broad abiotic and biotic gradients that have shaped their evolution. Gridded data for the long‐term average MAT and MAP for the reference period 1981–2010 were sourced from the CHELSA‐BIOCLIM dataset version 2.1 (Brun et al. 2022; Karger et al. 2017, Figures S3 and S4). P_avail_ was calculated for the 0–30 cm soil horizon, where soil P is most available to plants (Jobbágy and Jackson 2001), from the soil and landscape grid of Australia (Zund 2022, Figure S5).

Biotic environmental variables included the presence of flower‐visiting bats, the presence of flower‐visiting marsupials, and the species richness of flower‐visiting birds. The presence of flower‐visiting bats was calculated by overlaying all range maps from BatMap (Milne et al. 2023) for the eight species of bats known to regularly visit and forage from flowers in mainland Australia (table 7 in Armstrong 1979; Table S1). We used presence/absence rather than species richness due to the low number of flower‐visiting bat species in Australia and the large area of Australia with no flower‐visiting bats (Figure 1c). For similar reasons, we used presence/absence for flower‐visiting marsupials (Figure 1b), for which 14 species in genera *Acrobates*, *Cercartetus*, *Petaurus*, *Gymnobelideus*, and *Tarsipes* were selected from table 8 in Armstrong (1979) based on reports of nectar in their diet in Wilman et al. (2014) (Table S1). Species ranges for marsupials were from Marsh et al. (2022). Australian flower‐visiting birds, by comparison, are more diverse. Regular flower‐visiting birds include most of the 78 honeyeaters (Meliphagidae) as well as some parrots (Psittacidae), white‐eyes (Zosteropidae), *Cinnyris jugularis* (Nectariniidae) and others (table 6 in Armstrong 1979). We compiled a list of Australian bird species known to visit flowers and with nectar or pollen as part of their diet by cross‐referencing data from Garnett et al. (2015) and Wilman et al. (2014). We matched 92 flower‐visiting bird species (Table S1) to range maps from the Birds of the World version 2022.2 (BirdLife International and Handbook of the Birds of the World 2022), and overlaid these to calculate the species richness of flower‐visiting birds (Figure 1a).

We calculated the mean for each environmental variable across the range of each eucalypt species by extracting MAT, MAP, P_avail_, flower‐visiting bird species richness, flower‐visiting marsupial presence, and flower‐visiting bat presence at each occurrence record and averaging for each species. Most eucalypt species (bats *n* = 635, marsupials *n* = 590) had bats or marsupials either entirely present or entirely absent across their range (Figures S6 and S7). Remaining species were classified as bats present if bats occurred at more than 50% of species occurrences, and similarly for marsupials.

To produce trait maps we used eucalypt species ranges across Australia from Poisson point process modelling as reported in Gallagher et al. (2021). Evolutionary relationships were assessed using the Maximum Likelihood 1 phylogeny of Thornhill et al. (2019), a molecular phylogeny built from nuclear and plastid markers of 20 outgroup Myrtaceae species and 711 species of eucalypt (10 *Angophora*, 37 *Corymbia*, 664 *Eucalyptus*). Tree and range taxa were manually aligned to the species in our dataset.

### 2.3 Data Analysis

We tested for phylogenetic signal in flower size (a continuous trait) using Pagel's *λ* (Pagel 1999) and Blomberg et al.'s *K* (Blomberg et al. 2003) and in flower colourfulness (a binary trait) using caper (Orme et al. 2012) to calculate Fritz and Purvis' *D*, which is designed for categorical data (Fritz and Purvis 2010). We used phylogenetic generalised least squares (PGLS) regressions to assess two initial evolutionary hypotheses: (1) that larger eucalypt flower size and colourful flowers have evolved in correlation, and (2) that larger eucalypt flowers have evolved in correlation with lower median longitudes (i.e., more westerly distributions). We visualised the distribution of eucalypt flower size across the eucalypt phylogeny using the phytools function contMap (Revell 2024). We assumed evolution by Brownian motion to account for phylogenetic relatedness in trait analyses.

To test whether eucalypt flower size and colourfulness are better predicted by biotic or abiotic environmental variables, we built multiple regression models using the full set of predictors: flower‐visiting bird species richness, marsupial presence/absence, bat presence/absence (biotic), and MAT, MAP, P_avail_ (abiotic). Environmental variables were scaled to standardise effect sizes before analysis, and correlations among predictors were checked to avoid multicollinearity (Figure S8, correlations −0.42—0.72, Variance Inflation Factor < 4.2 in each case).

Flower size (a continuous variable) was analysed using OLS regression and PGLS regression to consider whether incorporating evolutionary history changed trait‐environment relationships. Flower colourfulness (a binary variable) was analysed using logistic regression and phylogenetic logistic regressions fit using the Ives and Garland Jr. (2010) method in phylolm v2.6.2 (Tung Ho and Ané 2014), though the latter showed negligible phylogenetic signal (*α*, which measures phylogenetic signal strength, ≅ 1), so we report non‐phylogenetic results.

To compare the predictive power of abiotic versus biotic factors, we fit three models for each trait: (1) a full model with all predictors, (2) a model with only biotic predictors, and (3) a model with only abiotic predictors. These models were compared using Bayesian Information Criterion (BIC), as it penalises model overfitting, and partial *R* ^2^, *R* ^2^_pred_, from Ives (2019) using the R package rr2 (Ives and Li 2018) to estimate variance explained. *R* ^2^_pred_ was chosen as it can be calculated for all models in our analysis and offers a direct measure of how much variation in data are explained by particular models (Ives 2019).

To assess the individual importance of each environmental variable, we performed model averaging across all possible predictor combinations with AICcmodavg v2.3‐2 (Mazerolle 2017), which provides standardised regression coefficients summarising each variable's contribution to the model (Burnham and Anderson 2002). We used model averaging on full flower size and flower colourfulness models.

## 3 Results

Eucalypt flowers are larger, on average, in southwestern Australia, and notably smaller along the southeast coast of Australia (Figure 2). Flower colour follows a similar pattern, with few eucalypts with colourful flowers found along the southeast coast of Australia and more colourful species in the centre to southwest (Figure S9).

Both flower size and flower colourfulness exhibit moderate phylogenetic signal (flower size Pagel's *λ* = 0.8, *p* < 0.001 and Blomberg's *K* = 0.02, *p* = 0.001; flower colourfulness Fritz and Purvis' *D* = 0.47, *p* < 0.001, Figure 3). Flower size and colourfulness are evolutionarily correlated, with larger eucalypt flowers more likely to be colourful (PGLS *p* < 0.001, df = 670, *R* ^2^_pred_ = 0.43, Figure 3, Figure S10). Flower size is also evolutionarily correlated with median longitude, with larger flowers more likely to evolve in species from western median longitudes (PGLS *p* = 0.005, df = 673, *R* ^2^_pred_ = 0.33, Figure S11). Larger, more colourful flowers appear to have evolved multiple times in western Australian eucalypts (Figure 3). For example, the clade of eucalypts with larger, colourful flowers at the base of section *Bisectae* is largely found in southwest Australia (e.g., *Eucalyptus rhodantha*, Figures 2 and 3), as are those at the end of section *Glandulosae* (e.g., *Eucalyptus tenera*, Figure 3). Several eastern Australian clades have predominantly small and white flowers, including a large part of subgenus *Eucalyptus* and subgenus *Symphyomyrtus* sections *Maidenaria*, *Exsertaria*, and *Adnataria* (Figure 3).

### 3.1 Predictors of Flower Size and Colour

Models containing biotic environmental predictors of eucalypt flower size and flower colourfulness had higher predictive power than models with only abiotic environmental predictors in all instances (Table 1, Table S2). For eucalypt flower size, a model with biotic predictors (bird richness, marsupial presence and bat presence) had the lowest BIC, outperforming the model combining abiotic and biotic predictors (ΔBIC = 2) and a model with abiotic predictors alone (ΔBIC = 17). The biotic model also explained more variance in eucalypt flower size (*R* ^2^_pred_ = 0.16) than the abiotic model (*R* ^2^_pred_ = 0.14), though a combined abiotic and biotic model explained the most variance overall (*R* ^2^_pred_ = 0.18, Table 1). Phylogenetic regression similarly showed the lowest BIC and highest *R* ^2^_pred_ for a biotic model, with higher *R* ^2^_pred_ overall (Table S2).

**TABLE 1:** Results from regression models used to compare the predictive power of abiotic versus biotic variables on eucalypt species mean flower size (log mm^2^) and species mean flower colour. Abiotic variables include eucalypt species mean annual temperature (°C), mean annual precipitation (mm) and available soil phosphorus (mg/kg). Biotic variables include eucalypt species mean flower‐visiting bird richness, marsupial presence/absence, and bat presence/absence. Models' predictive power was compared using Bayesian information criterion (BIC) and partial *R* ^2^_pred_ from Ives (2019).

Response variable	Model type	Predictor variables	BIC	*R* ^2^_pred_	Sample size
Flower size	Linear regression	Abiotic and biotic	2048	0.18	780
		**Biotic**	**2046**	0.16	780
		Abiotic	2062	0.14	780
Flower colour	Logistic regression	Abiotic and biotic	526	0.09	778
		**Biotic**	**514**	0.08	778
		Abiotic	526	0.06	778

*Note:* Models in bold have the lowest BIC.

Flower colourfulness followed a similar pattern where the model with the lowest BIC contained only biotic predictors, followed by abiotic and biotic combined (ΔBIC = 12) and abiotic predictors alone (ΔBIC = 12). Biotic variables alone explained slightly less variance in flower colourfulness (*R* ^2^_pred_ = 0.08) than the combined model (*R* ^2^_pred_ = 0.09), but more than the abiotic‐only model (*R* ^2^_pred_ = 0.06, Table 1), indicating that while biotic predictors offer the best model fit, abiotic predictors still contribute to explaining trait variation.

**FIGURE 4:**
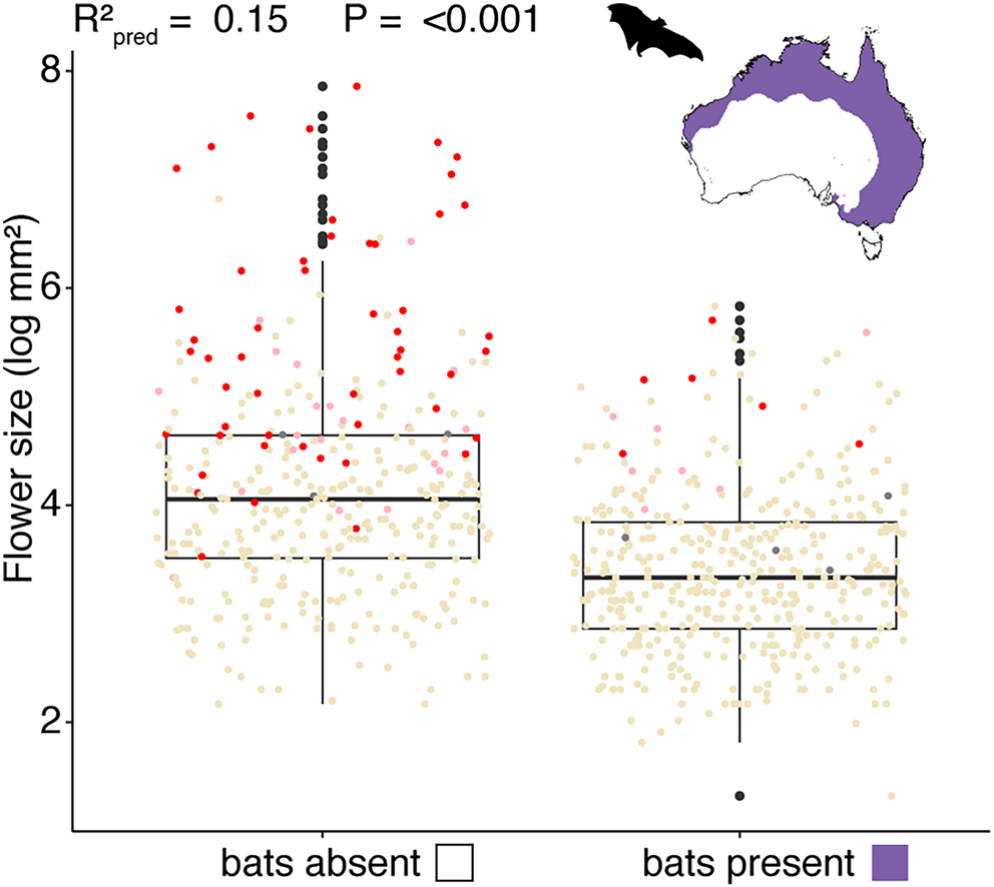
Boxplots of mean flower size (log mm^2^) for species with flower‐visiting bats absent or present in > 50% of their range. Individual species points are indicated by flower colour, with red points indicating colourful flowers, pink mixed, cream points indicating white‐cream flowers, and grey points indicating missing data. Map shows distribution (purple) of flower‐visiting bats across Australia, silhouette of *Pteropus poliocephalus* from phylopic.org. *R* ^2^_pred_ and *p*‐value from individual OLS regression.

Flower‐visiting bat presence was the strongest predictor of both eucalypt flower size and eucalypt flower colourfulness when the standardised regression coefficients of all environmental predictors were compared via model averaging (Table 2). Eucalypt flowers were generally larger and more colourful in environments without flower‐visiting bats, and bat presence/absence alone explained 15% of variation in eucalypt flower size (*R* ^2^_pred_ = 0.15; Figure 4). The second strongest predictor of eucalypt flower size from model averaging was available soil phosphorus, and for flower colourfulness, bird richness (Table 2). Eucalypt flowers tended to be bigger in environments with lower phosphorus, and more colourful with lower flower‐visiting bird richness (Table 2, Figure S12).

**TABLE 2:** Model‐averaged standardised regression coefficients for full eucalypt flower size and flower colourfulness models. *β* estimate column gives the coefficient (i.e., slope), S.E. gives standard error of this estimate. Higher absolute values of the coefficient indicate stronger relationships with the response variable.

Environmental predictor	Flower size		Flower colour	
	*β* estimate	SE	*β* estimate	SE
Mean annual temperature (°C)	0.05	0.05	0.34	0.24
Mean annual precipitation (mm)	0	0.04	−0.06	0.22
Available soil phosphorus (mg/kg)	−0.17	0.05	−0.42	0.22
Flower‐visiting bird richness	−0.02	0.05	−0.47	0.27
Flower‐visiting bat presence	−0.28	0.05	−0.85	0.32
Flower‐visiting marsupial presence	0.01	0.04	0.07	0.15

## 4 Discussion

Flower size and colour have evolved in tandem in the eucalypts, and larger, more colourful flowers are concentrated in southwest Australia (Figures 2 and 3, Figures S9–S11). The biotic pollination environment—in particular the presence/absence of flower‐visiting bats—is a stronger predictor of eucalypt flower size and colourfulness than is the abiotic environment (Tables 1 and 2). Continental‐scale patterns of floral trait variation thus reflect macroecological patterns in the availability of pollinators, revealing elements of the biotic environment that may shape the reproductive strategies of plants.

### 4.1 Pollination Environment

Of all the environmental predictors considered, bat presence had the strongest evidence of a relationship with flower size and colourfulness in eucalypts (Table 2). That is, flowers tend to be smaller and white‐cream in colour in environments with flower‐visiting bats present (Figure 4). Bats are effective outcross pollinators and can carry large pollen loads long distances between eucalypt trees (Hall and Richards 2000; Southerton et al. 2004). Bats often deposit more pollen on flowers than birds, perhaps because their fur can transport a larger quantity of pollen than bird feathers (Muchhala and Thomson 2010). Indeed, bats can have an outsized effect as eucalypt pollinators, accounting for a small proportion of pollinator visits but a large proportion of pollination due to the preferential outcrossing system of eucalypts (Bacles et al. 2009). Several floral features of some eucalypts have previously been suggested as adaptations for bat pollination, including mass flowering to attract nomadic bats, the production of large volumes of nectar at night, and the positioning of some eucalypt flowers in accessible terminal panicles (Hall and Richards 2000; Southerton et al. 2004). The white‐cream colour of most eucalypt flowers is more visible at night when bats are active, and small flower sizes allow numerous staminiferous flowers to be held closely together in an umbel, reminiscent of the “brush blossom” flower form common to bat pollination syndromes (Faegri and van der Pijl 1979; Fleming et al. 2009). Such flowers are also typically accessible to insect, bird, and marsupial pollinators and physically suitable for pollination by these groups, constituting a successful mixed pollination strategy for many eucalypts, with bats and birds both contributing to long‐distance pollen transfer (e.g., Bacles et al. 2009).

Though the importance of eucalypt flowers to pteropodid bats has long been recognised, the reciprocal importance of bats to eucalypts has received less attention in discussions of pollination. This may in part be because many studies of eucalypt pollination have been conducted in environments lacking flower‐visiting bats, in central and western Australia and Tasmania (e.g., Barber 1965; Bezemer et al. 2019; Bond and Brown 1979; Hingston et al. 2004a, 2004b; Hopper and Moran 1981; Kingston and McQuillan 2000; Savva et al. 1988). Indeed, pollination studies of eucalypts across northern Australia, where bats are likely most active as pollinators, are rare (House 1997). Even within the range of flower‐visiting bats, several eucalypt pollination studies have not considered the possibility of bat pollination, and conducted surveys only during the day (e.g., Ireland and Griffin 1984) or with methods that assess only insect pollinators (e.g., Griffin et al. 2009). Our finding that the presence of bats was the strongest environmental predictor of flower size and colourfulness in eucalypts suggests that bats may play a larger role in eucalypt reproduction than previously recognised. Bats should be considered a potentially important eucalypt pollinator in northern and eastern Australia, with experimental tests comparing the pollination effectiveness of bats, birds, insects, and marsupials to confirm this in more species.

In areas where flower‐visiting bats are absent, eucalypt flowers are more likely to be large and colourful, both adaptations that help attract bird pollinators, while large floral size may reduce the ability of smaller insect visitors to be effective pollinators (Faegri and van der Pijl 1979). In the absence of flower‐visiting bats, birds may be the next best option for eucalypts to ensure they transfer pollen long distances. This may be especially important for eucalypts that occur in small, isolated patches across the landscape, as has been shown, for example, with *Eucalyptus caesia* (Bezemer et al. 2016, 2019). Other traits may combine with flower size and colour to encourage bird pollination, such as flowering time and the volume and timing of nectar production. *Eucalyptus incrassata* , for instance, produces most nectar early in the morning, when birds are most actively foraging, and clusters its stamens tightly around the style such that insect pollinators have difficulty accessing the nectar (Bond and Brown 1979). Not all eucalypts in landscapes that lack bats have obvious bird pollination traits, however, and generalist bird and insect pollination is clearly at least sufficient for many eucalypts. Marsupial flower visitors may also play a role in pollination, though we found evidence of only a weak relationship between marsupial presence/absence and flower size or colourfulness (Table 2). This suggests that non‐flying mammals have not had a strong influence on the evolution of eucalypt flower traits, perhaps due to their limited pollen dispersal distance (House 1997). Experimental comparisons of mating systems, gene flow, and pollinator assemblages between closely related eucalypt pollination generalists and bird specialists could establish the factors favouring this evolutionary divergence, and whether the need for long‐distance pollen dispersal in a landscape without bat pollinators does indeed play a role.

Although we were unable to assess insect pollinator richness, abundance and activity due to data limitations, these factors may play a significant role in shaping the colour and size of eucalypt flowers. Although a broad range of pollinating insects are found in most parts of Australia, their activity likely varies considerably with temperature and rainfall (Ollerton 2017; Stephens et al. 2022). Indeed, *Eucalyptus urnigera* in temperate southeast Australia flowers in winter and receives very few visits from insect pollinators, instead favouring bird pollination, though its flowers remain white and small (Savva et al. 1988). Other, more subtle changes in insect pollinator assemblages may also shape eucalypt flower size and colour, such as the availability of native social bees across tropical northern Australia (Houston 2018) or that of predominantly fly‐pollinators at high altitudes (Kingston and McQuillan 2000). Unfortunately, current knowledge of the distributions of insect pollinators is too incomplete to assess these patterns at scale, as highlighted by our attempt to quantify the species richness of insect pollinators across Australia (Figure 1d, Note S1). Greater investment in the basic taxonomy and ecology of insect pollinators across Australia is urgently required (Braby 2018; Saunders et al. 2021).

### 4.2 Abiotic Environment

Though the abiotic environment was a weaker predictor of flower size and flower colourfulness in eucalypts than the pollination environment, it clearly has played some part. Of all abiotic factors, soil available phosphorus (P_avail_) was the strongest predictor of flower size and flower colour (Table 2). Surprisingly, eucalypt flowers were larger and more colourful in areas with low P_avail_ (Table 2, Figure S12), contrary to expectations from general patterns in leaf traits which are typically smaller in nutrient‐poor soils (e.g., Fonseca et al. 2000). Relationships between soil nutrients and flower size or colour are more complex, however, and may be mediated by pollinators and pollination syndromes (Carvalheiro et al. 2021). While flower size and colour are not themselves directly tied to nutrient content, they often co‐vary with other floral traits such as nectar volume and quality that influence pollinator attraction (Dellinger 2020; Fenster et al. 2004). Phosphorus limitation in Australia has been hypothesised to select for greater investment in vertebrate pollination through increased nectar rewards (Orians and Milewski 2007), though this remains largely untested. As yet little physiological evidence connects soil P to plant nectar production, though there is some evidence that plants increase root exudates under low soil P (Prescott et al. 2020). Soil nutrients can affect nectar and pollen quality, such as amino acid content, which in turn could influence pollination, but the strength and direction of these effects are yet to be established (Carvalheiro et al. 2021). Although eucalypt nectar and pollen are a significant part of the diets of many animals, few studies have quantified changes in their nutritional composition with abiotic environment. Our results suggest that nutrient limitation may play a role in shaping floral traits via pollination strategy, but more work is needed to uncover the underlying mechanisms.

Climate showed only weak relationships with flower size and flower colourfulness (Table 2). Flowers were marginally more colourful in higher MAT and lower MAP environments, perhaps reflecting the tendency of flower colours to be more saturated in more stressful environments (Dalrymple et al. 2020; Strauss and Whittall 2006). Neither MAT nor MAP had notable effects on eucalypt flower size, however, despite the effects gradients in these variables have on other plant and floral traits (Guerin et al. 2022; Lambrecht and Dawson 2007; Moles et al. 2014; Stephens et al. 2022). In light of our findings that pollination environment better predicts flower size, it seems likely that any effect of MAT or MAP on eucalypt flower size is obscured by differing and perhaps opposing pollination effects, as is common in a range of systems (Strauss and Whittall 2006). Both MAT and MAP, for example, have strong effects on flowering times across Australia, which in turn have strong effects on the distribution of flying‐foxes (Bradford et al. 2022; Hall and Richards 2000; Stephens et al. 2022). In this way, MAT and MAP may have complex, indirect effects on flower size and flower colourfulness in eucalypts mediated by the pollination environment.

### 4.3 Exploring Convergent Evolution, and Other Future Directions

Flower size and flower colourfulness have evolved in tandem in the eucalypts, and large, colourful flowers occur at multiple points in the eucalypt phylogeny, mostly associated with western Australian distributions (Figure 3, Figures S10 and S11). Large, colourful flowers have been linked to specialised bird pollination in several species (Bezemer et al. 2019; Hopper and Moran 1981; House 1997). It is possible that this is a case of convergent evolution, though extensive phylogenetic tests with a more robust phylogeny would be needed to confirm this. Eucalypt evolutionary history is an area of active research, and the phylogeny used here is one among several possible divergent scenarios (Crisp et al. 2024; Thornhill et al. 2019). Future studies could, for instance, confirm whether the small, white‐cream floral syndrome common to most eucalypt flowers is indeed ancestral for the clade, and if so, whether the multiple instances of large, colourful flowers represent more evolutionary transitions than would be expected by chance. This has been shown, for example, in the convergent evolution of flower colour in bird‐pollinated Australian angiosperms (Burd et al. 2014), and would represent a fascinating case of transition from generalist to specialist pollination syndromes. Surveys of changes in eucalypt floral traits and pollinator assemblage across the range of eucalypt sister species could also provide more detailed insight into the evolutionary drivers of shifts in floral traits, as has been shown, for example, in *Castilleja* (Hilpman and Busch 2021).

Detailed field surveys could also provide insight into drivers of eucalypt flower size and flower colour beyond the broad measures of environment considered here. In total, our measures of abiotic and biotic environment only explained 18% of variation in flower size (33% with phylogeny accounted for, Table S2), and 8% of variation in flower colourfulness (Table 1). Field measures could account for any intraspecific variability in flower size, flower colour, and environment not accounted for by our species means, though a field study would necessarily have a narrower taxonomic scope. A field study could also develop a more continuous measure of flower color beyond our broad colourful/not colourful human colour vision categories (e.g., as in Dalrymple et al. 2020), and account for polymorphic taxa. Future studies could consider the effect of environment on eucalypt fruit as well as flower size, given these traits are highly correlated in eucalypts (*R* ^2^_pred_ = 0.85, Note S2, Figure S13). Larger eucalypt fruits may be selected for in environments that experience more frequent or intense fires and lower rainfall (Murray and Gill 2001), potentially complicating the relationship between flower size and environment. Another open question is what role pteropodid bats have played in the evolution of eucalypt flowers, though answers to this may require greater knowledge of the timing of pteropodid arrival on the Australian continent (currently thought to be between a few and 55 million years ago, Westcott and McKeown 2014).

## 5 Conclusions

Our study provides preliminary evidence that flower size and flower colour in eucalypts is best predicted by the biotic pollination environment, and in particular, the absence of flower‐visiting bats. Continental‐scale patterns of floral trait variation thus reflect macroecological patterns in pollinator assemblages. Further explorations of floral trait variation at large scales will help us to build a more complete picture of the broad ecological and evolutionary forces shaping the plants around us.

## Author Contributions

**R. E. Stephens:** conceptualization (lead), data curation (lead), formal analysis (lead), investigation (lead), methodology (lead), project administration (lead), resources (lead), software (lead), validation (lead), visualization (lead), writing – original draft (lead), writing – review and editing (equal). **H. Sauquet:** conceptualization (supporting), methodology (supporting), supervision (equal), writing – review and editing (equal). **B. Laugier:** data curation (supporting), writing – review and editing (equal). **C. R. Gosper:** conceptualization (supporting), investigation (supporting), writing – review and editing (equal). **R. V. Gallagher:** conceptualization (supporting), investigation (supporting), methodology (supporting), supervision (equal), writing – review and editing (equal).

## Conflicts of Interest

The authors declare no conflicts of interest.

## Supporting information


Appendix S1.

## Data Availability

Full data, data processing, and analysis code are available at https://doi.org/10.5281/zenodo.10578823.
