# Supplementary material for: Birds, Bats or Climate? Eucalypt Floral Traits Reflect Pollination Over Abiotic Environment
Source: Ecol Evol. 2025 Jun 11;15(6):e71449. doi: 10.1002/ece3.71449 (PMC12152646; doi:10.1002/ece3.71449)
Supplement: Supplementary file 1 — Appendix S1. [file ECE3-15-e71449-s001.docx]

**Appendix S1: Supplementary figures and notes**

**Figure S1** Species richness of eucalypts (genera *Eucalyptus, Corymbia* and *Angophora*) in 100x100km grid cells across Australia, from species ranges modelled by Poisson point process modelling as reported in Gallagher et al. (2021).

**Note S1 – Flower-visiting insect species richness calculation**

To generate the map of flower-visiting insect species richness displayed in Figure 1d we first compiled a list of insect families known to act as pollinators in Australia from Armstrong (1979). A full list of insect clades and code used to generate map is available at https://github.com/rubysaltbush/flower-size-eucs/blob/main/scripts/figures/insect_pollinators_map.R. We then downloaded all occurrences for these species from the Atlas of Living Australia (ALA) using the R package galah v2.0.0 (Westgate et al., 2023). We used the ‘ALA’ filter profile to exclude absence-only records, eDNA and fossil records, records before 1700, duplicates, and records with uncertain coordinates, giving a total of 2,081,100 records from a combination of citizen science and museum specimen data. We used these records to summarise the species richness of flower-visiting insect species per 100x100 km grid cell across Australia, and display the result in Figure 1d. We used a log scale as some grid cells contain very high species richness (max = 5122), while most contained medium to low species richness (mean = 285, S.E. = 21.5). The patterns of species richness in Figure 1d strongly reflect sampling biases in Australia, with high species richness concentrated around the most populated Australian cities, where museums are also located, and low to no species richness in arid inland regions of Australia where the human population is very low and access is extremely difficult. This reflects the poor state of knowledge of insect species and their distributions in Australia, which is hampered especially by taxonomic shortfalls (Braby, 2018; Saunders et al., 2021).

**Figure S2** Ordinary least squares regression of eucalypt (*Eucalyptus, Corymbia* and *Angophora*) bud versus flower size (log mm^2^). Sizes are calculated as length (base of hypanthium to tip of gynoecium) × width (widest part across), as measured from a randomly selected bud and flower on a single eucalypt individual. *n* = 23 species.

**Figure S3** Australian mean annual air temperature (ºC) for 1981-2010 from CHELSA-BIOCLIM v2.1 (Brun et al., 2022; Karger et al., 2017).

**Figure S4** Australian mean annual precipitation (mm) for 1981-2010 from CHELSA-BIOCLIM v2.1 (Brun et al., 2022; Karger et al., 2017).

**Figure S5** Australian mean available soil phosphorus (mg/kg) in the top 30cm of soil derived from the Soil and Landscape Grid of Australia (Zund, 2022).

**Table S1** Flower-visiting Australian bat, marsupial and bird species included in calculations of flower-visiting bat, marsupial and bird species richness across Australia.

| **Group** | **Family** | **Species scientific name** | **Species common name** |
| --- | --- | --- | --- |
| Bats | Pteropodidae | *Dobsonia magna* | Bare-backed Fruit-bat |
|  |  | *Macroglossus minimus* | Northern Blossom Bat |
|  |  | *Nyctimene robinsoni* | Eastern Tube-nosed Bat |
|  |  | *Pteropus alecto* | Black Flying-fox |
|  |  | *Pteropus conspicillatus* | Spectacled Flying-fox |
|  |  | *Pteropus poliocephalus* | Grey-headed Flying Fox |
|  |  | *Pteropus scapulatus* | Little Red Flying-fox |
|  |  | *Syconycteris australis* | Eastern Blossom Bat |
| Marsupials | Acrobatidae | *Acrobates frontalis* | Broad-toed Feathertail Glider |
|  |  | *Acrobates pygmaeus* | Feathertail Glider |
|  | Burramyidae | *Cercartetus caudatus* | Long-tailed Pygmy Possum |
|  |  | *Cercartetus concinnus* | Western Pygmy Possum |
|  |  | *Cercartetus lepidus* | Little Pygmy Possum |
|  |  | *Cercartetus nanus* | Eastern Pygmy Possum |
|  | Petauridae | *Gymnobelideus leadbeateri* | Leadbeater's Possum |
|  |  | *Petaurus ariel* | Savanna Glider |
|  |  | *Petaurus australis* | Yellow-bellied Glider |
|  |  | *Petaurus breviceps* | Sugar Glider |
|  |  | *Petaurus gracilis* | Mahogany Glider |
|  |  | *Petaurus norfolcensis* | Squirrel Glider |
|  |  | *Petaurus notatus* | Krefft's Glider |
|  | Tarsipedidae | *Tarsipes rostratus* | Honey Possum |
| Birds | Artamidae | *Artamus cinereus* | Black-faced Woodswallow |
|  |  | *Artamus minor* | Little Woodswallow |
|  |  | *Artamus personatus* | Masked Woodswallow |
|  |  | *Artamus superciliosus* | White-browed Woodswallow |
|  | Campephagidae | *Lalage tricolor* | White-winged Triller |
|  | Climacteridae | *Climacteris picumnus* | Brown Treecreeper |
|  | Dicruridae | *Dicrurus bracteatus* | Spangled Drongo |
|  | Maluridae | *Amytornis merrotsyi* | Short-tailed Grasswren |
|  | Meliphagidae | *Acanthagenys rufogularis* | Spiny-cheeked Honeyeater |
|  |  | *Acanthorhynchus superciliosus* | Wet Tropics Eastern Spinebill |
|  |  | *Acanthorhynchus tenuirostris* | Eastern Spinebill |
|  |  | *Anthochaera carunculata* | Red Wattlebird |
|  |  | *Anthochaera chrysoptera* | Little Wattlebird |
|  |  | *Anthochaera lunulata* | Western Wattlebird |
|  |  | *Anthochaera paradoxa* | Yellow Wattlebird |
|  |  | *Anthochaera phrygia* | Regent Honeyeater |
|  |  | *Bolemoreus frenatus* | Bridled Honeyeater |
|  |  | *Bolemoreus hindwoodi* | Eungella Honeyeater |
|  |  | *Caligavis chrysops* | Yellow-faced Honeyeater |
|  |  | *Certhionyx variegatus* | Pied Honeyeater |
|  |  | *Cissomela pectoralis* | Banded Honeyeater |
|  |  | *Conopophila albogularis* | Rufous-banded Honeyeater |
|  |  | *Conopophila rufogularis* | Rufous-throated Honeyeater |
|  |  | *Conopophila whitei* | Grey Honeyeater |
|  |  | *Entomyzon cyanotis* | Blue-faced Honeyeater |
|  |  | *Epthianura tricolor* | Crimson Chat |
|  |  | *Gavicalis fasciogularis* | Mangrove Honeyeater |
|  |  | *Gavicalis versicolor* | Varied Honeyeater |
|  |  | *Gavicalis virescens* | Singing Honeyeater |
|  |  | *Gliciphila melanops* | Tawny-crowned Honeyeater |
|  |  | *Glycichaera fallax* | Green-backed Honeyeater |
|  |  | *Grantiella picta* | Painted Honeyeater |
|  |  | *Lichenostomus cratitius* | Purple-gaped Honeyeater |
|  |  | *Lichenostomus melanops* | Yellow-tufted Honeyeater |
|  |  | *Lichmera indistincta* | Brown Honeyeater |
|  |  | *Manorina flavigula* | Yellow-throated Miner |
|  |  | *Manorina melanocephala* | Noisy Miner |
|  |  | *Manorina melanophrys* | Bell Miner |
|  |  | *Manorina melanotis* | Black-eared Miner |
|  |  | *Meliphaga lewinii* | Lewin's Honeyeater |
|  |  | *Meliphaga notata* | Yellow-spotted Honeyeater |
|  |  | *Melithreptus affinis* | Black-headed Honeyeater |
|  |  | *Melithreptus albogularis* | White-throated Honeyeater |
|  |  | *Melithreptus brevirostris* | Brown-headed Honeyeater |
|  |  | *Melithreptus gularis* | Black-chinned Honeyeater |
|  |  | *Melithreptus lunatus* | White-naped Honeyeater |
|  |  | *Melithreptus validirostris* | Strong-billed Honeyeater |
|  |  | *Microptilotis albilineatus* | White-lined Honeyeater |
|  |  | *Microptilotis gracilis* | Graceful Honeyeater |
|  |  | *Myzomela erythrocephala* | Red-headed Honeyeater |
|  |  | *Myzomela obscura* | Dusky Honeyeater |
|  |  | *Myzomela sanguinolenta* | Scarlet Honeyeater |
|  |  | *Nesoptilotis flavicollis* | Yellow-throated Honeyeater |
|  |  | *Nesoptilotis leucotis* | White-eared Honeyeater |
|  |  | *Philemon argenticeps* | Silver-crowned Friarbird |
|  |  | *Philemon buceroides* | Helmeted Friarbird |
|  |  | *Philemon citreogularis* | Little Friarbird |
|  |  | *Philemon corniculatus* | Noisy Friarbird |
|  |  | *Phylidonyris niger* | White-cheeked Honeyeater |
|  |  | *Phylidonyris novaehollandiae* | New Holland Honeyeater |
|  |  | *Phylidonyris pyrrhopterus* | Crescent Honeyeater |
|  |  | *Plectorhyncha lanceolata* | Striped Honeyeater |
|  |  | *Ptilotula flavescens* | Yellow-tinted Honeyeater |
|  |  | *Ptilotula fusca* | Fuscous Honeyeater |
|  |  | *Ptilotula keartlandi* | Grey-headed Honeyeater |
|  |  | *Ptilotula ornata* | Yellow-plumed Honyeater |
|  |  | *Ptilotula penicillata* | White-plumed Honyeeater |
|  |  | *Ptilotula plumula* | Grey-fronted Honeyeater |
|  |  | *Purnella albifrons* | White-fronted Honeyeater |
|  |  | *Ramsayornis fasciatus* | Bar-breasted Honeyeater |
|  |  | *Ramsayornis modestus* | Brown-backed Honeyeater |
|  |  | *Stomiopera flava* | Yellow Honeyeater |
|  |  | *Stomiopera unicolor* | White-gaped Honeyeater |
|  |  | *Sugomel nigrum* | Black Honeyeater |
|  |  | *Trichodere cockerelli* | White-streaked Honeyeater |
|  |  | *Xanthotis flaviventer* | Tawny-breasted Honeyeater |
|  |  | *Xanthotis macleayanus* | Macleay's Honeyeater |
|  | Nectarinidae | *Cinnyris jugularis* | Olive-backed Sunbird |
|  | Psittaculidae | *Cyclopsitta diophthalma* | Double-eyed Fig Parrot |
|  |  | *Glossopsitta concinna* | Musk Lorikeet |
|  |  | *Glossopsitta porphyrocephala* | Purple-crowned Lorikeet |
|  |  | *Glossopsitta pusilla* | Little Lorikeet |
|  |  | *Lathamus discolor* | Swift Parrot |
|  |  | *Platycercus venustus* | Northern Rosella |
|  |  | *Polytelis alexandrae* | Princess Parrot |
|  |  | *Psitteuteles versicolor* | Varied Lorikeet |
|  |  | *Trichoglossus chlorolepidotus* | Scaly-breasted Lorikeet |
|  |  | *Trichoglossus moluccanus* | Rainbow Lorikeet |
|  | Ptilonorhynchidae | *Ptilonorhynchus violaceus* | Satin Bowerbird |
|  |  | *Sericulus chrysocephalus* | Regent Bowerbird |
|  | Zosteropidae | *Zosterops lateralis* | Silvereye |
|  |  | *Zosterops luteus* | Yellow White-eye |

**Figure S6** Histogram showing the number of eucalypt species found entirely within the range of flower-visiting bats (1), entirely outside of the range of flower-visiting bats (0), or with some of their modelled range inside and some outside the range of flower-visiting bats (>0 and <1).

**Figure S7** Histogram showing the number of eucalypt species found entirely within the range of flower-visiting marsupials (1), entirely outside of the range of flower-visiting marsupials (0), or with some of their modelled range inside and some outside the range of flower-visiting marsupials (>0 and <1).

**Figure S8** Pairwise correlation plot for all biotic and abiotic environmental predictors included in multiple regressions.

**Figure S9** Mean eucalypt flower colour in 100 x 100 km grid cells across Australia. Cell values were calculated by averaging for all eucalypt species that occur in that grid cell according to their modelled ranges whether they have colourful (1) or white-cream flowers (0).

**Figure S10** Mean eucalypt flower size (log mm^2^) by flower colour. Flower size and flower colour are evolutionarily correlated, with PGLS *p* < 0.001, R^2^_pred_ = 0.43.

**Figure S11** Mean eucalypt flower size (log mm^2^) by eucalypt species median longitudes, coloured by flower colour. Lower median longitudes correspond to more westerly distributions. The line is drawn from a phylogenetic generalised least squares regression (PGLS) with *p* = 0.005, R^2^_pred_ = 0.33.

**Table S2** Results from phylogenetic regression models used to compare the predictive power of abiotic versus biotic variables on eucalypt species mean flower size (log mm^2^). Abiotic variables include eucalypt species mean Mean Annual Temperature (ºC), Mean Annual Precipitation (mm) and available soil phosphorus (mg/kg). Biotic variables include eucalypt species mean flower-visiting bird richness, flower-visiting marsupial presence/absence and flower-visiting bat presence/absence. Models’ predictive power was compared using Bayesian Information Criterion (BIC) and partial R^2^_pred_ from Ives (2019).

| **Response variable** | **Model type** | **Predictor variables** | **BIC** | **R^2^_pred_** | **Sample size** |
| --- | --- | --- | --- | --- | --- |
| Flower size | PGLS | Abiotic and biotic | 2201 | 0.330 | 675 |
|  |  | **Biotic** | **2190** | 0.331 | 675 |
|  |  | Abiotic | 2204 | 0.326 | 675 |


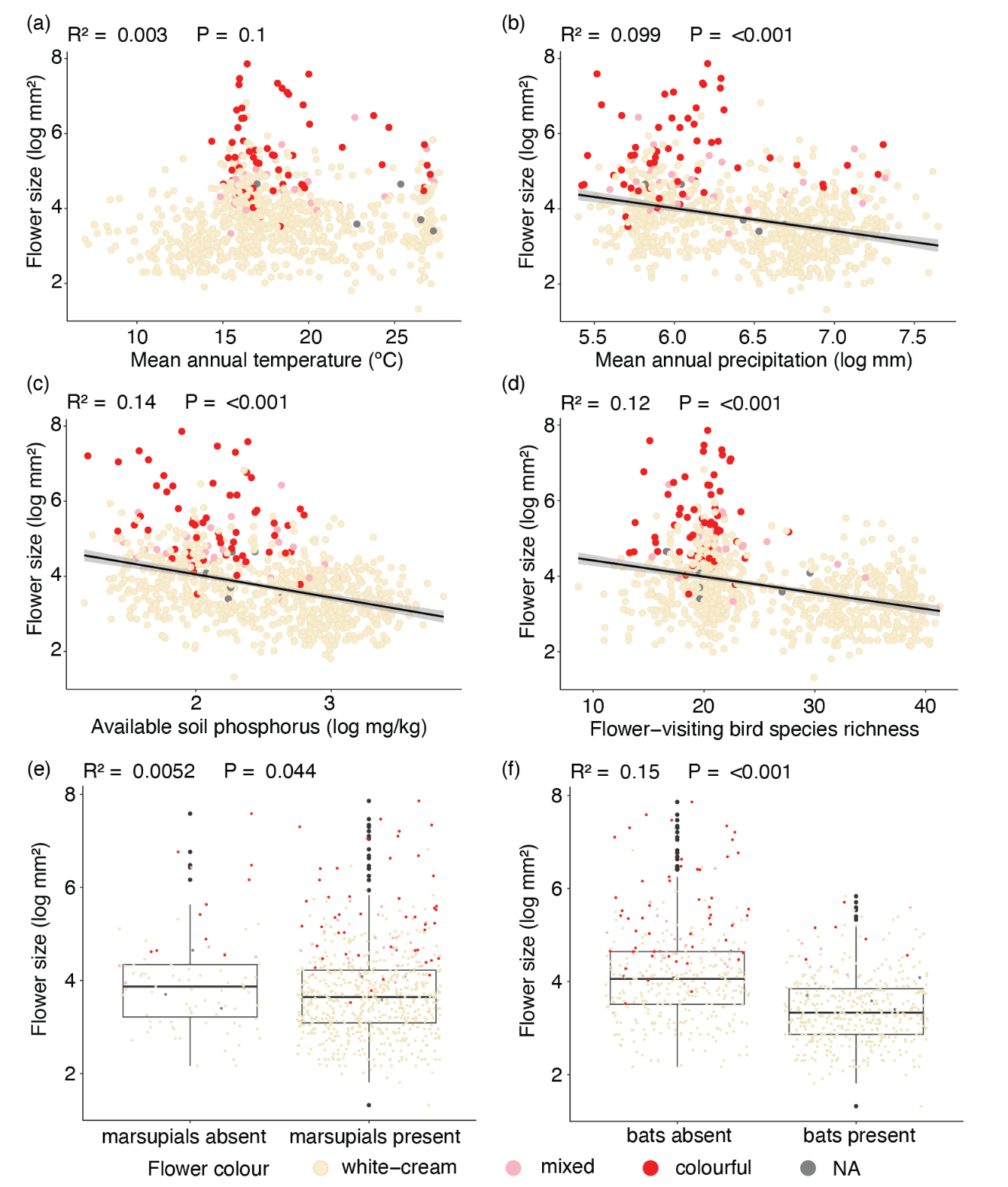


**Figure S12** Eucalypt species mean flower size (log mm^2^) individual relationships with abiotic (a – mean annual temperature, ºC; b – mean annual precipitation, mm; c – mean available phosphorus, log mg/kg) and biotic (d – flower-visiting bird species richness, e – flower-visiting marsupial presence/absence, f – flower-visiting bat presence/absence) environmental predictors. R^2^ and p-values from individual OLS regression. Note that these relationships change when all environmental predictors are considered together in multiple regressions, see main text Table 2 for model-averaged standardised effect size for each predictor.

**Note S2 – Eucalypt fruit size and bud size are highly correlated**

Reproductive traits are often tightly correlated in plants. To confirm whether our proxy for flower size, bud size, was strongly correlated with fruit size in eucalypts we used similar methods to extract fruit dimensions from EUCLID (CANBR et al., 2020). As for bud size species mean fruit size in mm^2^ was calculated from fruit dimensions by taking the mean of minimum and maximum values for length and width, then multiplying length × width. Both bud size and fruit size were log-transformed and their relationship assessed via phylogenetic least squares regression (PGLS) for n = 678 species. This showed that fruit size and bud size are highly correlated, with R^2^_pred_ = 0.85 and *p* < 0.001 (Figure S13). Any environmental selection for bud or flower size may thus be complicated by environmental selection for fruit size, as these traits are highly coordinated in eucalypts.

**Figure S13** Phylogenetic generalised least squares regression (PGLS) predicting eucalypt bud size (log mm^2^) by eucalypt fruit size (log mm^2^), *p* < 0.001, d.f. = 676, *R^2^*_pred_ = 0.85.
